# Phyloanatomic characterization of the distinct T cell and monocyte contributions to the peripheral blood HIV population within the host

**DOI:** 10.1093/ve/veaa005

**Published:** 2020-04-27

**Authors:** Brittany RifeMagalis, Samantha L Strickland, Stephen D Shank, Patrick Autissier, Alexandra Schuetz, Pasiri Sithinamsuwan, Sukalaya Lerdlum, James L K Fletcher, Mark de Souza, Jintanat Ananworanich, Victor Valcour, The Search007 Study Group, Kenneth C Williams, Sergei L Kosakovsky Pond, Silvia RattoKim, Marco Salemi

**Affiliations:** v1 Department of Pathology, Immunology, and Laboratory Medicine, Emerging Pathogens Institute, University of Florida, Gainesville, FL 32601, USA; v2 Department of Biology, Temple University, Philadelphia, PA 19122, USA; v3 Department of Biology, Boston College, Chestnut Hill, MA 02467, USA; v4 Department of Retrovirology, Armed Forces Research Institute of Medical Sciences - United States Component, Bangkok 10400, Thailand; v5 SEARCH, Thai Red Cross AIDS Research Center, Bangkok 10330, Thailand; v6 U.S. Military HIV Research Program, Walter Reed Army Institute of Research, Rockville, MD 20850, USA; v7 Division of Neurology, Department of Medicine, Phramongkutklao Hospital, Bangkok 10400, Thailand; v8 Faculty of Medicine, Department of Radiology, Chulalongkorn University, Bangkok 10330, Thailand; v9 Department of Neurology, University of California San Francisco, San Francisco, CA 94143, USA

**Keywords:** HIV, evolution, phyloanatomy, monocyte, T cell

## Abstract

Human immunodeficiency virus (HIV) is a rapidly evolving virus, allowing its genetic sequence to act as a fingerprint for epidemiological processes among, as well as within, individual infected hosts. Though primarily infecting the CD4+ T-cell population, HIV can also be found in monocytes, an immune cell population that differs in several aspects from the canonical T-cell viral target. Using single genome viral sequencing and statistical phylogenetic inference, we investigated the viral RNA diversity and relative contribution of each of these immune cell types to the viral population within the peripheral blood. Results provide evidence of an increased prevalence of circulating monocytes harboring virus in individuals with high viral load in the absence of suppressive antiretroviral therapy. Bayesian phyloanatomic analysis of three of these individuals demonstrated a measurable role for these cells, but not the circulating T-cell population, as a source of cell-free virus in the plasma, supporting the hypothesis that these cells can act as an additional conduit of virus spread.

## 1. Introduction

The low reported frequency (<0.1%) of human immunodeficiency virus (HIV)-infected monocytes in the peripheral blood (Sonza et al. 2001) and the difficulty of obtaining tissue samples present major barriers to elucidating the dynamics of HIV infection in these cells (Sonza et al. 2001; Honeycutt et al. 2016). Earlier studies focusing on the majority population of circulating monocytes (CD14++) identified viral population and genetic differences from that of T cells (Zhu et al. 2002; Fulcher et al. 2004; Delobel et al. 2005; Llewellyn et al. 2006), consistent with the hypothesis of cell-specific viral population dynamics. Investigation of the role(s) of monocytes in the evolution and spread of HIV-1 has been largely limited to the differentiated macrophage phenotype (Marsden and Zack 2008), complicating interpretations owing to the ability of the latter to acquire virus not only from the bone marrow or peripheral blood as undifferentiated monocytes but also from the resident tissue or by ingestion of infected cells (Tacke et al. 2006; Calantone et al. 2014; Sattentau and Stevenson 2016). Difficulties in isolating viral RNA from patient-derived CD14++ monocytes (Wang et al. 2013), owing to low susceptibility to infection in comparison to the CD16+ population (Ellery et al. 2007) as well as potential defective integrated virus, have also impeded the inclusion of monocytes in models of evolutionary trajectories among differing anatomical compartments (Frost et al. 2005; Cybis et al. 2013; Lorenzo-Redondo et al. 2016; Salemi and Rife 2016). In this study, we demonstrate the successful isolation of viral RNA from several monocyte subpopulations for use in a phyloanatomic study (Salemi and Rife 2016) of the evolutionary dynamics and genetic features of HIV in the peripheral blood of a subset of CRF01_AE-infected individuals. The findings of this study reveal a measurable role for circulating monocytes as a source of viral diversity and gene flow within the blood of individuals with high viral load (VL). 

## 2. Materials and methods

### 2.1 Ethics statement

Fifty HIV-1-infected, combination antiretroviral therapy (cART)-naive volunteers were enrolled into the SEARCH 007 study (registration number NCT00777426) at the Thai Red Cross AIDS Research Center in Bangkok, Thailand (Ratto-Kim et al. 2018). Hepatitis C infection and illicit drug use within the past 5 years as well as a positive illicit drug screen were exclusion criteria (further information can be found under clinicaltrials.gov). All volunteers met the Thai Ministry of Health guidelines to initiate therapy based on having symptomatic HIV infection or a CD4+ T-cell count below 350 cells/ml (Valcour et al. 2013). Signed informed consent was obtained for all participants, consisting of adults at least 18 years of age. The study was approved by the Chulalongkorn University Institutional Review Board in Bangkok, Thailand, and the University of Hawaii.

HIV+ volunteers initiated therapy (zidovudine or stavudine, lamivudine, and nevirapine) at time of enrollment and returned to the clinic at 3-month intervals for the duration of 1 year. Peripheral mononuclear cells (PBMCs) were collected and neuropsychological testing performed at baseline and 12 months following enrollment/cART initiation. Data were generated for the study from baseline and at 12 months post-cART initiation.

### 2.2 Cell sorting

Cryopreserved PBMCs were quickly thawed in a 37 °C water bath before being transferred to a 50 ml conical tube containing 40 ml RPMI with 20 per cent FBS pre-warmed at 37 °C. Cells were washed twice and transferred to an FACS tube and stained for 15 min at room temperature with an antibody cocktail consisting of anti-CD14-Pacific Blue (clone M5E2), anti-CD3-Alexa Fluor 700 (clone SP34-2), anti-CD20-Cy7-APC (clone B27) and anti-CD16-Cy7-PE (clone 3G8) (all from BD Pharmingen, San Jose, CA), anti-HLA-DR-ECD (clone L243, Beckman Coulter, Miami, FL), and Live/Dead Aqua (Invitrogen, Eugene, OR, USA). All antibodies were titrated to determine optimal concentrations. Antibody-capture beads (CompBeads, BD Biosciences) were used for single-color compensation controls for each reagent used in the study, with the exception of cells being used for anti-CD3 and Live/Dead Aqua. After staining, cells were washed once, filtered, and resuspended in 1 ml PBS. The BD FACSAria cytometer (BD Biosciences, San Jose, CA, USA) was set up with a pressure of 20 psi and a 100-μm nozzle was used. Instrument calibration was checked daily by use of rainbow fluorescent particles (BD Biosciences). After acquiring unstained and single-color control samples to calculate the compensation matrix, we acquired 1 × 10^6^ events in order to set up the sorting gating strategy. CD14+ monocyte population were gated first based on FSC and SSC parameters, after which we excluded (1) dead cells by gating out Aqua+ cells and (2) unwanted cells by gating out CD3+ and CD20+ cells and then gated on HLA-DR+ cells. From the HLA-DR+ population, a dot plot of CD14 versus CD16 was used to make a sorting gate, which included all monocytes except the CD14–CD16− subset. For CD3+ T-lymphocyte sorting, FSC and SSC parameters were used to gate lymphocytes, dead cells were excluded by using Aqua staining, and CD14+ cells were also excluded. Following this procedure, the CD3+ T-lymphocytes were gated based on CD3 expression and negativity for CD16. Postsort purity were checked for each sample, and both CD14+ and CD3+ sorted subpopulations were >98 per cent pure. After cell sorting, the highly pure cell populations were washed with PBS twice and all liquid was aspirated. Cells were then stored as dry pellets at −80 °C.

### 2.3 RNA extraction and cDNA synthesis

Cell-free viral RNA was extracted from participant plasma using the Qiagen QIAamp Viral RNA Mini Kit, whereas sorted PBMC-associated RNA and DNA were processed using the Qiagen Allprep DNA/RNA Mini kit according to the manufacturer’s protocols. Viral RNA was then reverse transcribed into cDNA according to the manufacturer’s protocol using the SuperScript^®^ III First-Strand Synthesis System kit (Invitrogen). The following primer was used for reverse transcription: ‘K-env-R1’ 5′-CCAATCAGGGAAGAAGCCTTG-3′ (HXB2 coordinates 8736-8716) (Utachee et al. 2009).

### 2.4 Single genome amplification and sequencing

HIV-1 env *gp120* sequences were amplified from viral cDNA and genomic DNA using a modified limiting-dilution two-round PCR approach [‘single genome sequencing’ (SGS)] based on previously published methods (Palmer et al. 2005) in order to prevent PCR-mediated resampling and recombination. The following primers were used for both rounds of PCR: ‘polenv_AE’ 5′-GAGCAGAAGACAGTGGAAATGA-3′ (HXB2 coordinates 6207–6228; modified from Tuttle et al. (2002) for subtype AE) and ‘192H’ 5′-CCATAGTGCTTCCTGCTGCT-3′ (HXB2 coordinates 7815–7796; modified from Maureen Goodenow for subtype AE). PCR reactions consisted of 2 min at 94 °C for 1 cycle, 30 s at 94 °C, 30 s at 58 °C, and 3 min at 72 °C for forty cycles, then 10 min at 72 °C using the Platinum^®^ Blue PCR SuperMix (Invitrogen). Amplicons were then visualized using 1 per cent agarose gel electrophoresis with an Amplisize™ Molecular Ruler 50–2,000 base pair (bp) ladder (Bio-Rad). Sequencing was performed using an Applied Biosystems 3730xl DNA Analyzer (Life Technologies) at the University of Florida Interdisciplinary Center for Biotechnology Research genomics core facility. 

RNA and DNA extractions, cDNA synthesis, and first-round polymerase chain reaction (PCR) set-up were conducted in a restricted-access, amplicon-free room with separate air-handling, with laboratory equipment where no amplified PCR products or recombinant cloned plasmids were allowed, and where work surfaces and equipment were thoroughly cleaned before and after use with Eliminase^®^ (Decon Labs, Inc.). PCR loading was performed so as to minimize contamination across plasma and cell-specific samples for individual participants. For example, PCR amplification plate no. 162 contained diluted RNA from P01V1 and P02V1, but only for monocytes. RNA sequences were used for phylogenetic analysis so as to mitigate the potential effects of defective proviral DNA on evolutionary inferences.

### 2.5 Sequence alignment and analysis

Individual RNA nucleotide sequence chromatograms were visualized using Geneious vR6 (Kearse et al. 2012) for the investigation of sites assigned multiple nucleotide identities and identification of potential sequencing errors. Artifacts induced during single genome amplification (including insertions, deletions, and misincorporations) amount to a rate of ∼8×10^–^^5^ (Salazar-Gonzalez et al. 2008). We have also accounted for errors incorporated during Sanger sequencing, found to be approximately 1 bp per 100 sequences using this region in Strickland et al. (2011). Consequently, a conservative approach was used to remove single nucleotide insertions or deletions (replaced with majority nucleotide) to ensure reliable interpretation of phylogenetic relationships. Sequences are available in GenBank (MK842155–MK843233). Patient-specific sequences were translated and aligned using the Clustal algorithm (Thompson et al. 1997) implemented in BioEdit v7.1.11 (Hall 1999) followed by manual optimization of positional homology (Lamers, Sleasman, and Goodenow 1996) and removal of gap-filled regions within the hypervariable V1V2 domains. The final alignment included 1,220 nucleotides spanning position 6381–7601 of the HXB2 reference strain. Six of the total 332 T-cell sequences (1.8%) contained stop codons, similar to the frequency (2.0%) in monocytes (2/90), and greater than that of plasma (0.15%, ratio of 1/657), as would be expected given plasma virus is cell-free and represents a population of replication-competent virus. As each of the individual premature stop codon substitutions were singleton misincorporations within the population at that site, however, they likely represent sequencing errors, rather than functionally defective GP120, and were substituted with an ambiguous nucleotide (‘N’) for selection analysis. Putative intra-host recombinants were identified using the Phi test in SplitsTree4 (Huson and Bryant 2006) and removed prior to phylogenetic analysis. Alignments are available from thai.hyphy.org. Neighbor-joining (NJ) tree reconstruction was then performed using MEGA v5.2.2 (Tamura et al. 2011) with the HKY model of nucleotide substitution (Hasegawa, Kishino, and Yano 1985) and gamma-distributed rate variation across sites. Pairwise deletion was used for treatment of gaps within the alignment. Branch support was assessed by bootstrapping (1,000 replicates). Sequences from all participants were included in the NJ tree in order to infer participant viral subtype and the extent of sequencing cross-contamination based on participant-specific clustering patterns (Supplementary Fig. S1).

Evolutionary analysis was performed for participants (P01, P02, and P13) from whom greater than three sequences were obtained from each of the three compartments (plasma, monocytes, and T cells). Sequences from two separate time points (0 and 12 months) were analyzed for P01, who did not suppress viral replication, despite the initiation of cART upon enrollment. Viral genetic diversity was quantified by pairwise genetic distances estimated for sequences derived from cell-free virus in the plasma and from sorted peripheral T-lymphocytes and monocytes within the three previously described individuals (P01, P02, and 013). This estimation was performed in R (APE package) (Paradis, Claude, and Strimmer 2004) using the TN93 nucleotide substitution model (Tamura and Nei 1993) with gamma distributed rate variation across sites (*α *= 0.1). A viral epidemiology signature pattern analysis was used to detect distinct frequency variation in particular amino acids between plasma, T-lymphocyte, and monocyte viral sequences for P01, P02, and P13, respectively.

### 2.6 Maximum likelihood tree reconstruction and compartmentalization analysis

Because viral population structure, such as that dependent on anatomical location or cell type, can affect patterns of polymorphism that contribute to significant genetic variation or that mimic selection (Pannell and Charlesworth 2000), the extent of this structure was assessed both qualitatively and quantitatively within each participant. Maximum likelihood (ML) tree reconstruction was performed for each of the three individuals using all available *gp120* sequences in order to assess clustering patterns according to anatomical location and time of sampling and was performed in IQ-Tree (Nguyen et al. 2015) using the best-fit evolutionary model according to the Bayesian Information Criterion. Tree correlation coefficients were estimated to provide a quantitative assessment of compartmentalization, representing the relationship between population isolation and the distance within the tree. Population subdivision was defined in terms of anatomical sampling origin (as well as time for P01), and the extent of isolation was measured according to the number of branches (*r*_b_) or patristic distances (*r*) separating two sequences (Critchlow et al. 2000; Zarate et al. 2007). Anatomical population structure was analyzed in order to also determine if within-host epidemiological linkages between peripheral cell populations and plasma could be resolved with confidence, as uncertainty regarding epidemiological origins increases with significant compartmentalization (De Maio et al. 2015).

### 2.7 Selection analysis

Selection analyses were performed using a modification of the fixed effects likelihood (FEL) method for estimating site-specific selective pressures (Kosakovsky Pond and Frost 2005). The modified test, dubbed contrast-FEL (or cFEL), is available in HYPHY (Pond, Frost, and Muse 2005), v2.5.0 or later. To use cFEL, we partitioned each intra-host ML tree containing all available sequences for each individual into groups of branches according to their compartment membership, and assigned labels for internal branches if and only if all of their descendants belonged to the same compartment. This procedure defined four sets of branches: Plasma, T cell, Monocyte, and background (i.e. not labeled). We next fitted the MG94xREV model to the entire alignment by ML to estimate nucleotide substitution biases and relative branch lengths. Next, for every site, we fitted a site-specific MG94xREV model with five parameters: *α* (site-wide synonymous rate, relative to the gene average), and four compartment-specific nonsynonymous substitution rates *β_K_*, where K∈plasma,T cell, monocyte, background. A site was designated as showing evidence of differential selection if two conditions were met. First, there was a significant likelihood ratio (LR) test (LRT) for different *β_K_* rates, either overall, or between any pair of rates; *P*-values were derived using $\chi^{2}$ asymptotic test distributions subject to Holm–Bonferonni correction for multiple testing at the site. Second, the value of the LR statistic for the given branch labeling was not exceeded by LR values of any of the 20 site-level ‘permutation’ tests, where branch labels were randomly shuffled for each test and the same set of tests was carried out using thus relabeled branches. Annotated trees and alignments for patients P01, P02, and P13, along with cFEL results and mapped cFEL-identified sites on the 3D Env structure (3JWO) (Pancera et al. 2010), are available at thai.hyphy.org and exemplified in (Supplementary Fig. S2).

### 2.8 Bayesian phyloanatomic reconstruction

Because sampling was not uniform across sampling locations for these three participants, three replicates of random sampling (without replacement) according to the minimal number of available sequences in one of the three locations (P001 = 15, P002 = 20, P013 = 10) was performed for each of the three participants (with equal representation of months 0 and 12 for P01). This approach acts to reduce the impact of spatial sampling bias in the Bayesian phylogenetic analysis while incorporating more information from the larger sequence populations than would be available from a single random sample. Phylogenetic signal was determined prior to Bayesian analysis for each of the participant-specific sample replicates using likelihood mapping (Strimmer and von Haeseler 1997) implemented in TreePuzzle v5.2 (Schmidt et al. 2002), the results of which indicated sufficient signal for phylogenetic analysis (Supplementary Fig. S3). Temporal signal was also assessed for individual sample replicates of P01, consisting of sequences sampled at multiple time points, by determining the significance of the relationship of sequence sampling time to genetic divergence from the most recent common ancestor of all sequences within the transmission cluster. We used a clustered permutation approach in BEAST v1.8.3 (Drummond et al. 2005, 2012, asking whether the correlation was stronger than expected if sampling dates were randomly assigned among clusters of sequences sampled on the same date (Duchene et al. 2015). Clustered permutation tip date randomization (Duchene et al. 2015; Murray et al. 2016) was performed in R using the TipDatingBeast package for five replicates (Rieux and Khatchikian 2017) and used for Bayesian evolutionary reconstruction in BEAST assuming the uncorrelated, relaxed molecular clock (Drummond et al. 2006), and Bayesian skyline demographic models (Drummond et al. 2005). Markov chain Monte Carlo sampling of parameters and tree topologies was performed for 500 million generations or until effective sample sizes (ESS) reached values >200 (after burn-in of 10%). ESS were calculated in Tracer (Rambaut et al. 2018). A diffusion model allowing for asymmetric transition rates among discrete anatomical locations (Edwards et al. 2011) was incorporated into the nonrandomized tip date BEAST analysis, as migration rates are assumed to be independent of evolutionary reconstruction (i.e. no impact on dating). The Bayesian stochastic search variable selection (BSSVS) procedure (Lemey et al. 2009) allowed for the identification of significantly nonzero, nonreversible rates (i.e. directionality) of viral dispersion between sampled anatomical compartments (plasma, T-lymphocytes, and monocytes). A continuous-time MC-induced counting process was implemented to log-labeled transition events in time (Minin and Suchard 2007, 2008). The hierarchical phylogenetic model (HPM) was used to summarize trends across the three sample replicates for each participant (Edo-Matas et al. 2011). As mentioned previously, because monocyte sequences were unobtainable at later sampling time points for P002 and P013, only sequences from the first visit (V1) for these two participants were used for phyloanatomic analysis. Given the robustness of the molecular clock for contemporaneously sampled HIV sequences (Park et al. 2016), relaxed clock calibration was enforced with a prior mean evolutionary rate of 6.88E−04 substitutions/site/month, based on previous estimates (Lemey, Rambaut, and Pybus 2006). A similar HPM study was implemented in Cybis et al. (2013) to summarize gene flow between immune cell compartments across patients, finding between 0.05 and 0.1 per cent of the stratum (model of gene flow for each patient) edges differed from their counterparts in the hierarchical graph (model of gene flow across patients). As multiple random samples from within a single patient are expected to exhibit less variation than between host samples, we chose the lower bound of this range (0.05) as the cutoff for the HPM prior probability for an edge in each of the three stratum graphs for P01 to differ from the hierarchical graph of P01. Detailed information regarding additional evolutionary parameters and associated priors used in BEAST analysis is available from GitHub (see Data Availability section). Trees sampled (1,000) from the posterior distribution (after burn-in of 10%) were visualized simultaneously and branch density assessed using DensiTree (Bouckaert 2010), with high-density areas indicative of increased certainty of clustering patterns.

### 2.9 Statistical analysis

Significant differences for compartment-specific viral diversity (pairwise genetic distance) between participants were determined using a nonparametric pairwise multiple comparisons analysis based on rank sums (Dunn test package in R) with Bonferroni *P*-value correction following rejection of the D’Agostino Pearson test of normality. A *P*-value of ≤0.05 was considered significant. Statistical significance for Critchlow’s correlation coefficient (Slatkin 1989) was determined using a null distribution of permuted sequences (1,000 permutations). A *P*-value of ≥0.05 was considered significant. For the tip date randomization test, absence of overlap between 95 per cent high posterior density intervals of the mean evolutionary rate of the randomized and the correctly dated run indicated significant temporal signal (Supplementary Fig. S4). With regard to the BSSVS analysis, Bayes factors (BF) were calculated according to Lemey et al. (2009) for participant-specific transition rates between each compartment using a truncated Poisson prior (50% prior probability on the minimal rate configuration). Transition rates with BF >3 were considered to be well supported (Lemey et al. 2009). For the cFEL analysis, a *P*-value of ≤0.05 was considered significant.

## 3. Results

### 3.1 Successful amplification of monocyte-derived viral RNA is achieved with high VL

Peripheral blood samples were obtained from individuals (CD4 count <350 cells/mm^3^) enrolled in a volunteer longitudinal HIV study in Thailand (Ratto-Kim et al. 2018) Using sorting techniques relying on immune-specific cluster of differentiation (CD) cell surface markers, we isolated, with ≥98 per cent purity, monocyte and T-cell populations (Supplementary Fig. S5) in thirteen of twenty-two individuals (Table 1). We generated envelope glycoprotein *gp120* monocyte-derived RNA sequences in ten of the participants using SGS (Palmer et al. 2005; Supplementary Fig. S6), though positive RNA levels below the assay limit of detection cannot be ruled out for the remaining individuals. Higher VLs were positively correlated with the normalized number of estimated monocyte-derived viral RNA copies ($R^{2}=0.4$, Supplementary Fig. S7) and were marginally predictive of the ability to successfully sequence monocyte-derived viral RNA (*P *=* *0.06, logistic regression, Supplementary Fig. S8). In three of the participants (P01, P02, and P13) with VL >10^5^ copies/ml (Table 1 and Supplementary Fig. S8), we successfully generated 10–48 *gp120* sequences from each of the peripheral monocyte, T cell, and plasma locations, permitting more robust phylogenetic inference, as methods such as population structure detection are sensitive to sampling bias (Zarate et al. 2007). Patient P01 did not successfully achieve viral suppression at 12 months following initiation of cART, suggesting nonadherence to therapy, though time of interruption is unknown (Table 1).

**Table 1.** Viral burden in plasma and sorted PBMCs among Thai cohort.

Participant	Time since first visit (cART initiation)					
	0 months (V1)			12 months (V5)		
	Viral load (copies/ml)	Mono HIV RNA genomes^a^	SE	Viral load (copies/ml)	Mono HIV RNA genomes^a^	SE
P01^b^	750,000	269,875	27,639	100,000	129,484	3,6435
P02^b^	397,150	156,548	30,127	263	ND	–
P03	96,396	6,773	2,709	<50	ND	–
P04	183,154	24,680	10,212	<50	ND	–
P07	46,885	3,434	1,919	<50	ND	–
P08	16,616	1,677	1,572	<50	ND	–
P13^b^	540,909	12,038	3,398	<50	ND	–
P15	350,439	5,422	3,030	<50	ND	–
P19	98,808	4,037	2,663	<50	ND	–
P21	385,478	4,698	2,181	<50	ND	–
P24	232,930	5,314	1,811	<50	ND	–
P26	99,649	ND	–	<50	ND	–
P29	17,277	6,793	2,445	<50	ND	–

^a^ Copies per million monocytes and standard error (SE) as estimated in QUALITY (Rodrigo et al. 1997) using limiting dilution PCR results, unless not detectable (ND).

^b^ Participants from which HIV genomic RNA was PCR-amplified (bold) and 10 or more sequences per cell type were successfully obtained.

### 3.2 Monocytes represent ancestral origins in the intra-host genealogies of infected individuals

**Figure 1.**
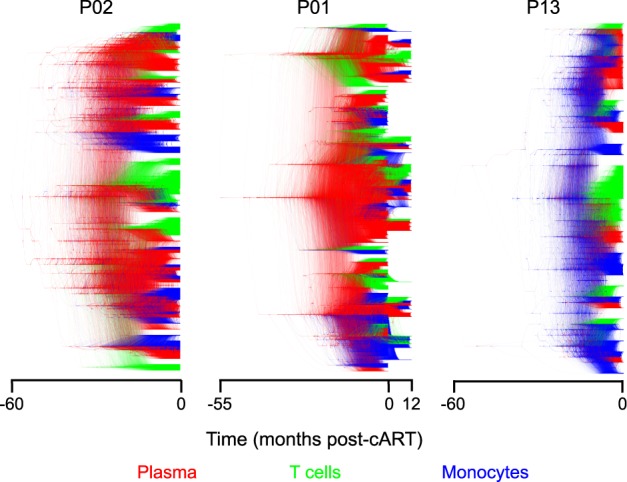
Sampled posterior distribution of Bayesian phylogenetic trees for all HIV gp120 sequences derived from plasma and sorted peripheral leukocytes in three Thai individuals. A sample (1,000) from the posterior distribution was obtained using a relaxed molecular clock model of evolutionary rate variation across branches (Drummond et al. 2006) and constant population size over time. Branch lengths are scaled in time and colored according to sampling origin (see legend along bottom). Well-supported branches are indicated by solid colors whereas webs represent little agreement.

We performed a spatiotemporal reconstruction of the evolutionary history of virus from plasma and blood cell populations using a Bayesian phyloanatomic framework (Lemey et al. 2009; Salemi and Rife 2016). A varying proportion of ancestral lineages were inferred to be of monocyte origin for all three individuals (Fig. 1), with the strongest signal for participant P13. Monocyte viral lineages did not form a monophyletic clade in the time-scaled phylogenies (Fig. 1), or the divergence-scaled ML phylogenies (Supplementary Fig. S9), for any of the three individuals, a finding that does not support a model of completely evolutionarily separate cellular compartments, or significant population structure. We sought to validate this conclusion using quantitative analyses of the pairwise genetic distances between origin-annotated sequences within the ML trees (Supplementary Table S1), which similarly did not support a genetically structured environment. Because of the relatively sparse sampling of intra-host populations it is not possible to make definitive claims regarding the cellular source of any particular viral subpopulation. However, recent models developed in the context of between host HIV source attribution (Romero-Severson, Bulla, and Leitner 2016) leverage the topological structure of viral phylogenies to infer that Compartment A (e.g.) is likely the source of virus in Compartment B if multiple clades of virus from B are nested within the larger clade of virus from A. Although uncertainty in the timing of the origin of all sampled sequences is represented by the relatively low-density branching in the most interior portions of the patient phylogeny samples (Fig. 1), the high-density paraphyletic clustering suggested a high degree of confidence for inferences regarding the source of virus among the sampled anatomical locations. It is clear from the solidly colored backbones of each tree distribution that plasma (red) would be considered a measurable source of virus in P01 and P02, whereas monocytes (blue) would play a more prominent role in P13. Tree distributions for each patient have been summarized in the form of a maximum clade credibility tree in (Supplementary Fig. S10).

### 3.3 Monocytes represent a measurable source of plasma virus in the peripheral blood

**Figure 2.**
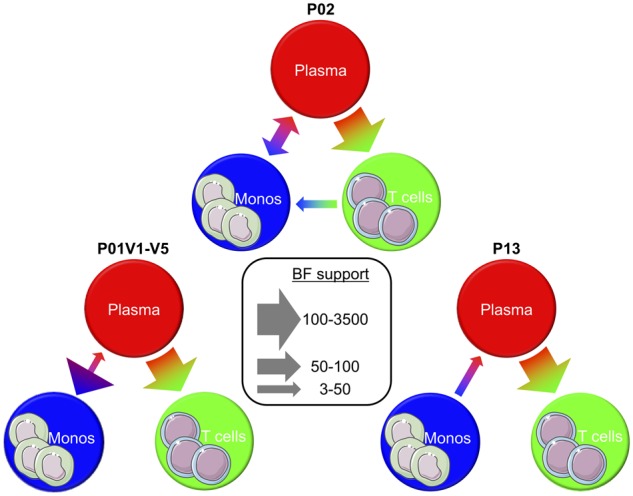
Inferred viral dispersion among peripheral blood compartments in three HIV-1-infected Thai individuals with sufficient samples. BEAST v1.8.3 (Drummond and Rambaut 2007; Drummond et al. 2012) was used to reconstruct *gp120* evolutionary histories for each of the three participants (P01, P02, and P13) and to model migration according to an asymmetric diffusion process among discrete anatomical locations (Edwards et al. 2011). Statistically significant migration rates were identified conditional on having BF >3 (Lemey et al. 2009) and are depicted as arrows with widths proportional to the BF (legend in center).

As spatiotemporal inferences using the coalescent framework can be sensitive to sampling bias (Hall, Woolhouse, and Rambaut 2016), sequences from the three individuals with relatively high VL were randomly sub-sampled to mitigate compartment sampling bias for statistical phyloanatomy analysis. Furthermore, as variation has been observed between replicates of the same sampling scheme (Hall, Woolhouse, and Rambaut 2016), a hierarchical model was used to incorporate data across sub-samples (see Section 2). The role of the monocyte ancestral lineages in viral dispersion within the peripheral blood for each of the three individuals was assessed using BSSVS under an asymmetric diffusion model (Fig. 2). We found a statistically significant contribution of monocytes to cell-free viral lineages in the plasma of all individuals, despite differences in sampling time intervals among the individuals, in agreement with earlier studies (Zhu et al. 2002). Similarly, the contribution of plasma virus to peripheral T-cell lineages was supported in all individuals (BF >3; Lemey et al. 2009). The three participants differed only in the contribution of plasma and peripheral T cells to viral genetic dissemination in monocytes, with contribution from both populations in P02, plasma virus alone in P01, and neither population in P13.

### 3.4 Viral gene flow among PBMCs and plasma is most prominent prior to ART

**Figure 3.**
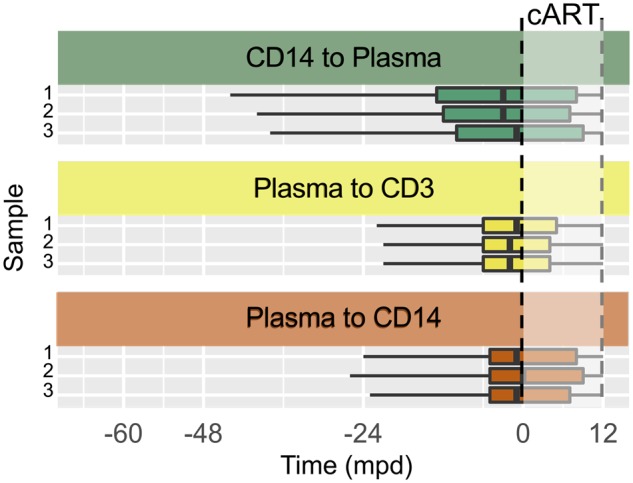
Inferred timing of viral dispersion among discrete anatomical compartments for participant P01 sample replicates. Participant *gp120* sequence data were re-sampled thrice (with replacement) according to the minimum number of sequences in one of the three anatomical compartments. The timing, in months postdiagnosis (mpd), of viral dispersion was inferred for individual P01 sample replicates (one to three) using the Bayesian phyloanatomy (Salemi and Rife 2016) framework and counts derived using the CTMC counting process described in Minin and Suchard (2007, 2008). *Bayes factor support (BF >3) indicating a significantly nonzero rate of transition between designated discrete anatomical locations within the Bayesian phylogeny, as determined using the BSSVS model (Lemey et al. 2009) of asymmetric transition rates.

Relatively large monocyte sequence sample size at the time of enrollment and 12-month follow-up for P01 offered an opportunity for molecular clock dating of specific gene flow events. However, as the patient was being treated during at least a portion of this time, measures were taken to confirm viral divergence from the initial viral sample and, thus, reliable temporal inferences (Drummond et al. 2003; Rambaut et al. 2016). Analysis of time-dependent phylogenetic clustering of sequences revealed statistically significant genetic distinction between the two different time points (Supplementary Table S1), indicating viral population turnover and sufficient evolution during the sampling time interval. Date randomization tests (Duchene et al. 2015; Murray et al. 2016; Rieux and Khatchikian 2017) were also performed to determine if greater evolutionary change was occurring over the 12-month period than would be expected by random sampling of available dates, confirming measurable evolution between sampling time points (Supplementary Fig. S4). Temporal reconstruction in P01 indicated that the median time of transmission for each of the well-supported transitions occurred prior to administration of cART, although extending into the first year post-therapy in this individual (Fig. 3). Despite potential sampling variation that can accompany the spatial sampling strategy described for these individuals, temporal inferences of the well-supported dispersion patterns in P01 overlapped by as much as 100 per cent among the three replicates of sequence sub-sampling (Fig. 3), indicating the robustness of the molecular clock analysis and BSSVS approach. Timing of each of the well-supported gene flow events suggests an inhibitory influence of prolonged cART exposure on viral dispersion, though not completely inhibitory.

### 3.5 Peripheral blood compartments differ in GP120 genetic diversity and site-specific selection pressure

We observed statistically significant differences in mean pairwise nucleotide diversity (per cent) between monocyte-derived *gp120* RNA sequences and those of plasma and T cells (Supplementary Fig. S11). This finding is in agreement with earlier studies using viral DNA (Fulcher et al. 2004; Llewellyn et al. 2006), though patterns differed among patients and sampling times. In P02, HIV isolates sampled from monocytes (2.1%) were significantly less diverse (*P *<* *0.001) than those from plasma (3.2%) and T cells (5.0%). In P13, monocyte HIV diversity (2.4%) was significantly greater (P* *<* *0.001) than that in plasma (1.4%), but not significantly different from that in T cells (2.8%). In P01 at enrollment, the pattern agreed with P02—HIV in monocytes (2.9%) was significantly (P* *<* *0.0001) less diverse than either plasma (3.1%) or T cells (5.0%). At 12 months post-cART, however, the pattern was reversed—HIV in monocytes (4.3%) was significantly (P* *<* *0.01) more diverse than both plasma (3.2%) and T cells (4.1%). Statistically significant differences and variation in the relative levels of genetic diversity suggested diverging evolutionary processes and/or selection pressure among peripheral blood compartments, though patient-specific factors cannot be excluded. In particular, the time of infection for each patient is unknown.

**Figure 4.**
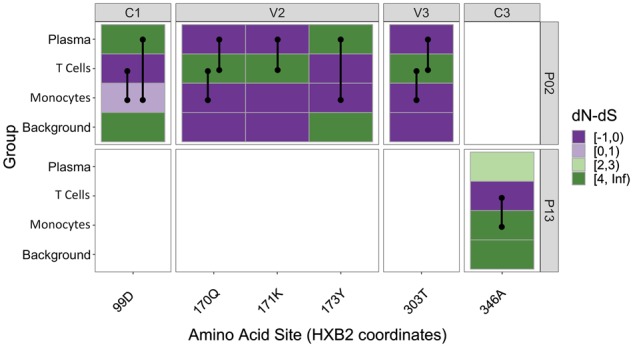
GP120 sites reporting statistically significant differences in selective pressure between branch classifications for each Thai individual. Branches within patient-specific maximum likelihood trees were classified as foreground—plasma, T cells, or monocytes—or background (remaining internal branches). Each population-site pair is colored according to the difference in the rate of nonsynonymous (d*N*) and synonymous (d*S*) substitutions scaled by the total branch length accompanying site-specific changes. Amino acids comprising constant (C) and variable (V) loops, as defined previously (Leonard et al. 1990), are separated accordingly, with site numbers corresponding to the sequence alignments (aligned between all three patients). Sites reported to differ between any of the three foreground populations are indicated with a black line drawn between the respective population pairs. NA blocks for amino acid site 269 of the P13 sequence alignment indicate no statistically significant overall difference when including background branches. Sites are numbered according to the HXB2 reference sequence coordinates. P-values ≤0.05 were considered significant.

Nonsynonymous nucleotide differences between virus from plasma, T cells, and monocytes were readily apparent upon assessment of amino acid composition, varying among both constant (C) and variable (V) regions of GP120 (Supplementary Fig. S12). A site-specific selection analysis was, therefore, performed in order to determine the extent of selection pressure associated with these differences. A population-level approach to site-specific selection detection was developed by adapting the previous d*N*/d*S*-based FEL model for LR testing (Kosakovsky Pond and Frost 2005) across population-designated branches (foreground) within the patient-specific ML trees. Given the statistically significant differences in diversity across sampling origins, we anticipated the identification of selected sites differentiating these populations in each individual but sought to specifically assess whether the number of sites experiencing selection or specific region within *gp120* was associated with differing viral gene flow patterns across individuals (Fig. 4). For P02, five sites were identified as differing significantly (P≤0.05) between a specific pair of sampling populations (e.g. monocytes vs. T cells). Three out of the five sites were located within the V2 region; however, they were not restricted to any specific pair of populations. Three out of the five sites were also categorized as differentiating monocyte and T-cell lineages, though these sites were not localized to any particular subregion (i.e. sites were distributed across the C1, V2, and V3 regions). The level of selection also varied according to site, though monocyte sequences exhibited d*N* −d*S* values <1 for all significant sites in this individual. d*N*−d*S* values varied across the board for plasma, T cell, and background lineages. Alternatively, in P13, one site was identified, located within the C3 region (no overlap with P02). No differing sites were reported for P01. The results suggest that the differences observed in genetic diversity and amino acid composition between anatomical populations were attributed at least in part to differences in selection pressure at individual amino acids sites. However, there was no clear role for the number of selected sites or region-specific selection pressure in the distinct gene flow patterns observed in each of the three individuals. Additional evidence is required to determine if sites differing in the extent of selection pressure between monocytes and T cells for two of the three patients is indicative of a distinct pressure associated with infection of monocytes, given their distinct locations within the primary sequence and small number of individuals.

## 4. Discussion

The blood of HIV-infected patients harbors a diverse viral population with potentially many phenotypes (Xu et al. 2008). Though CD4+ T cells are the primary target of the virus, circulating monocytes can harbor viral DNA populations genetically distinct from that of T cells (Zhu et al. 2002; Fulcher et al. 2004; Delobel et al. 2005; Llewellyn et al. 2006). The contribution of monocytes to viral replication and evolution within the host remains controversial, however, owing to concerns regarding the bias of defective integrated virus in evolutionary analysis (Bruner et al. 2016) and difficulty in RNA sequence isolation (Wang et al. 2013) from an already low-frequency infected cell population (<0.1%) (Sonza et al. 2001). In light of this controversy, the contribution of this cell population to viral spread within the peripheral blood, as well as remaining infected tissues, has yet to be investigated. We have successfully shown that HIV monocyte RNA can be detected at low levels (<7,000 copies/10^6^ cells) in the vast majority (12/13) of infected individuals using an expanded cell sorting strategy to include a wider range of monocyte subpopulations, followed by single genome amplification. Sequencing of viral RNA from these cells in addition to plasma and CD4+ T cells paved the way for sophisticated statistical analyses of viral genetic and epidemiological patterns within the peripheral blood.

In contrast with previous works (Fulcher et al. 2004; Delobel et al. 2005; Llewellyn et al. 2006), statistically significant population structure was not characteristic of PBMCs in the three infected individuals, potentially explained by the combined result of inclusion of the more susceptible monocyte subpopulation (CD16+) to HIV infection (Ellery et al. 2007) and an advantage of RNA over DNA in phylogenetic analysis. For example, greater prevalence of defective virus in either of the two PBMC compartments owing to clonal expansion would inflate within-population sequence similarity and may result in significant overestimation of the level of compartmentalization. Although *ex vivo* quantification of productive infection has indicated greater numbers of replication-competent virus in T cells than monocytes in SIV-infected macaques (Avalos et al. 2016), investigation of the fraction of the total viral DNA burden that is defective has not been undertaken, to our knowledge, nor the contribution of clonal expansion to these numbers. The absence of significant spatial genetic structure signaled, however, that the tempo and mode of gene flow among the sampled blood populations could be reliably inferred using the Bayesian phyloanatomy framework(Lorenzo-Redondo et al. 2016; Salemi and Rife 2016). Taking care to mitigate the effects of sampling bias and arbitrary phylogenetic rooting on estimates of the rate and directionality of gene flow events, we employed a Bayesian HPM and molecular clock rooting for spatially subsampled longitudinal data. Though viral RNA from all three sampling locations was detectable post-cART for only one of the individuals within the cohort, the role of monocytes as a statistically significant source of cell-free virus in the peripheral blood of this individual was consistent with that of cross-sectional (pre-cART only) data from the remaining two high VL individuals. This finding suggests that molecular clock rooting using serially sampled data were not necessary to address the question of monocyte origins over a 1-year infection duration, though a rigorous testing of data at both joint and individual time points among a larger subject population is required to test this hypothesis.

As an extension of an originally epidemiological concept, the findings of the Bayesian phyloanatomy analysis demonstrate that frequency of infection may lead to skewed perceptions of the respective roles of minority and majority populations in not only viral evolution but spread. In contrast to monocytes, T cells did not contribute statistically significantly to the plasma viral population in the peripheral blood. Although seemingly contradictory to what is known regarding HIV infection dynamics (CD4+ T cells are the primary cellular target), the majority of T cells in circulation are quiescent, producing lower levels of virus than their activated tissue counterparts (Zack, Kim, and Vatakis 2013). Additional information as to the behavior of the individual cell types (e.g. migration and location of activation) would, as in traditional epidemiological surveillance, aid in understanding the transmission dynamics of individual cell types. The measurable role of monocytes in peripheral blood HIV dynamics may also be explained by the long lifespan of monocyte virus (Zhu et al. 2002), owing to the resistance of these cells to the cytopathic effects of both the virus and antigen-specific immune response (Le Douce et al. 2010; Kumar, Abbas, and Herbein 2014). A long lifespan would result in a mix of ancient and contemporaneous viral lineages within the respective sample and thus greater diversity, explaining this observation for monocytes at the later time point in P01 and in Llewellyn et al. (2006). This increasing viral diversity over time in P01, and increased diversity relative to the surrounding peripheral blood in both P01 and P13, would provide a natural source of adaptive evolution within the host, even at the low level of replication typically associated with this cell population (Le Douce et al. 2010). However, it is also possible that contemporaneous sampling of significantly older viral variants results in over-confidence in reconstruction of evolutionary relationships, and thus origins, in the deeper lineages within the tree. Additional investigation of the impact of differences in the duration of infection of individual infected cell populations on branching patterns within the tree is needed to disentangle viable source contributions from biases.

One particular cautionary tale to phyloanatomy studies in the HIV-infected host is the uncertainty regarding virus origin, even within the cell. Owing to restrictions placed on viral reverse transcription in monocytes and quiescent T cells (Triques and Stevenson 2004; Bergamaschi and Pancino 2010), amplification of RNA alone does not provide definitive evidence of infection of these cell populations and may provide a false intermediate state in reconstruction of gene flow events in the context of evolution. *In vivo* evidence suggests that even viral DNA cannot be considered an accurate proxy for the infection of differentiated monocytes, as it can be obtained via phagocytic infection (Calantone et al. 2014), although this mechanism has been shown to improve efficiency of infection (Baxter et al. 2014). Given the uncertainty in true anatomical origin of the viral RNA sequenced in this study, it is not possible to assign 100 per cent probability for either of the two compartments to the sampled leaves within the patient genealogies. An approach that allows for trait uncertainty along a minority of these leaves would provide an additional estimate of the error involved, but we are unaware of such an approach, and it would be impractical to do so for all sequences from both or even a single cell population. However, it stands to reason that if viral RNA found within monocytes was the result of T-cell ingestion, gene flow would best be explained by T-cell contribution to monocytes, which was only observed for one of the three individuals. Rather, movement of virus from plasma to monocytes (which would be characterized by cell-free transmission) was supported in all three individuals. Additionally, RNA presence via ingestion would be expected to be represented by similar genetic composition between monocytes and T cells and thus equivalent rates of gene flow to the plasma. In contrast, statistical support was only identified for the flow of virus to plasma from monocytes, and was the case in not one, but all three infected individuals. Combined, these results suggest that the phagocytic behavior of macrophages may not similarly explain the presence of viral RNA in their circulating, and phenotypically distinct, precursors—the monocytes. This hypothesis is further supported by differing selection pressure, though the influence of selection on inferences of gene flow cannot be ruled out. A high level of selection pressure representing an enhanced ability to infect one compartment over the other, for example, would be expected to inflate the appearance of spatial structure, thus biasing gene flow rates inferred from the tree. In this study, there was no clear relationship outlined between the level of selection pressure and gene flow, as the individual with the largest number of selected sites showed the highest level of mixing among compartments (P02). Advanced sequencing techniques allowing for reconstruction of phylogenetic relationships using primarily neutral sites (e.g. third codon site in non-RNA-structured regions) without loss of phylogenetic signal (i.e. longer genomic regions; Bruner et al. 2016; Hiener et al. 2017) would aid in disentangling the stories of gene flow and selection.

Though a measurable contributor to viral spread in the absence of therapy, the difficulty in obtaining monocyte sequences following compliant therapy implies the ability of ART to inhibit viral uptake by this cell population (Almodovar et al. 2007) and its contributions to viral dissemination within the peripheral blood. The results are consistent with the common recognition of latently infected T cells as the predominant reservoir and contributor to viral rebound during treatment interruption (e.g. Rothenberger et al. 2015; Lu et al. 2018). However, more extensive sampling of infected tissues in the SIV-macaque model has revealed latent viral genomes with the potential for reactivation in both circulating monocytes and tissue-resident macrophages in virally suppressed animals (Abreu et al. 2019a,b). Their resilience to therapeutic interventions (Le Douce et al. 2010; Kumar, Abbas, and Herbein 2014), as well as migratory nature (Williams and Burdo 2012; Jakubzick, Randolph, and Henson 2017, Jakubzick et al. 2013), suggest monocytes should not be entirely ruled out as a source of viral persistence and continuing source of virus spread in the face of ART. It is important to note, however, the limited patient sample size associated with this particular type of study, present investigation included, and the need for a larger population study to accurately resolve the role of individual cell populations as reservoirs.

Limitation of this study to the peripheral blood compartment has impeded, yet encouraged, knowledge of the role of monocyte-rich tissues (e.g. bone marrow and draining lymph nodes) (Jakubzick, Randolph, and Henson 2017, Jakubzick et al. 2013), in viral dissemination. This limitation may explain the low power to detect a statistically significant original source of monocyte virus in P13 and recipients of T-cell virus in P01 and P13. Inclusion of additional tissues known to harbor differing monocyte and T-cell subpopulations (e.g. quiescent and activated) would allow for improved inferences of the connections between anatomical sources of viral diversity, migration, and selection pressure, and of the differences between HIV dynamics in the peripheral blood and deeper tissues.

## Data availability

The authors declare that all data supporting the findings of this study are available within the paper (and Supplementary data), but original data that supports the findings are available from GenBank (MK842155–MK843233), thai.hyphy.org, and https://github.com/brmagalis/HIV_THAI_COHORT.

## Supplementary Material

veaa005_Supplementary_Data
