# Supplementary figures and images for: Phyloanatomic characterization of the distinct T cell and monocyte contributions to the peripheral blood HIV population within the host
Source: Virus Evol. 2020 Apr 27;6(1):veaa005. doi: 10.1093/ve/veaa005 (PMC7185683; doi:10.1093/ve/veaa005)

P02

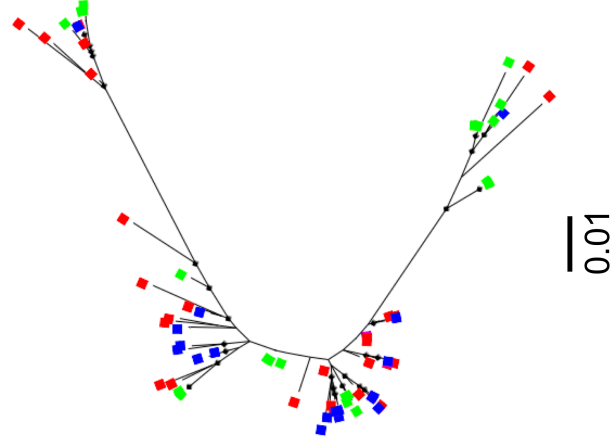

P01

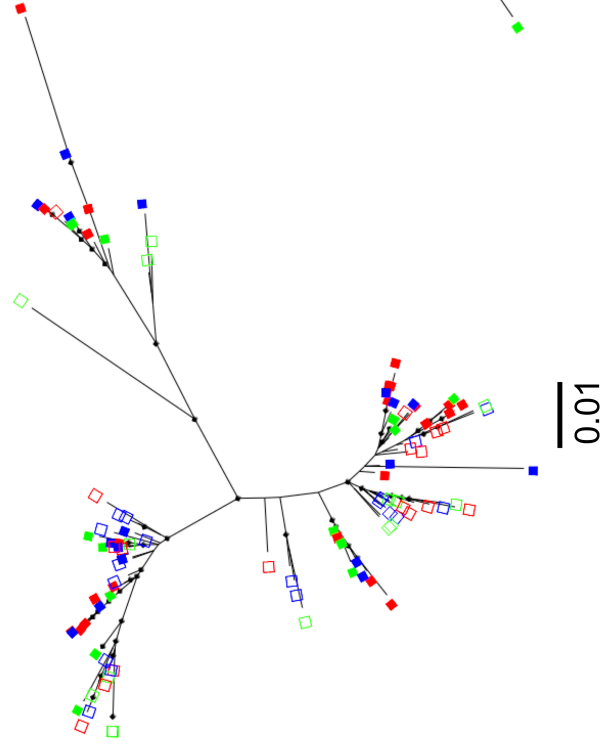

P13

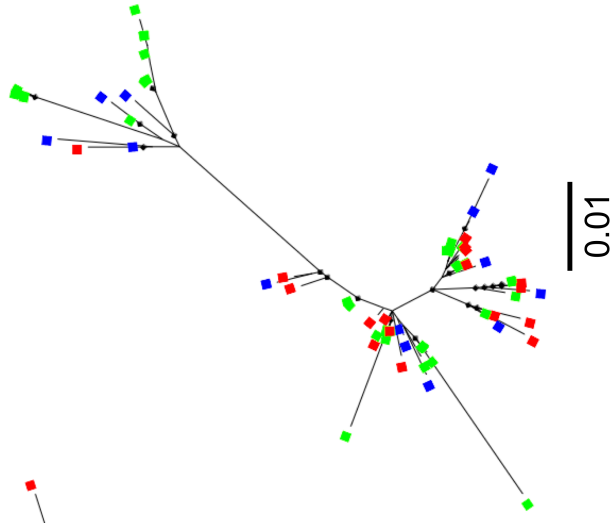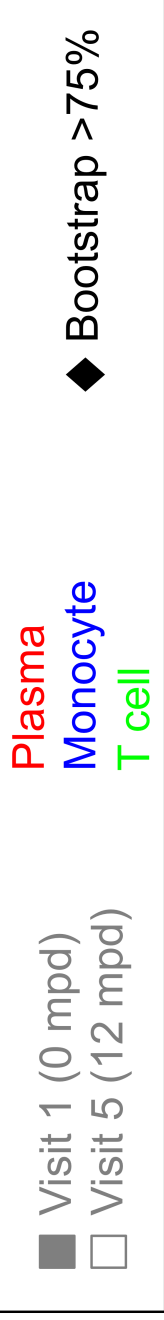

Supplement: veaa005_Supplementary_Data [file veaa005_supplementary_data.zip › FigS9.pdf]

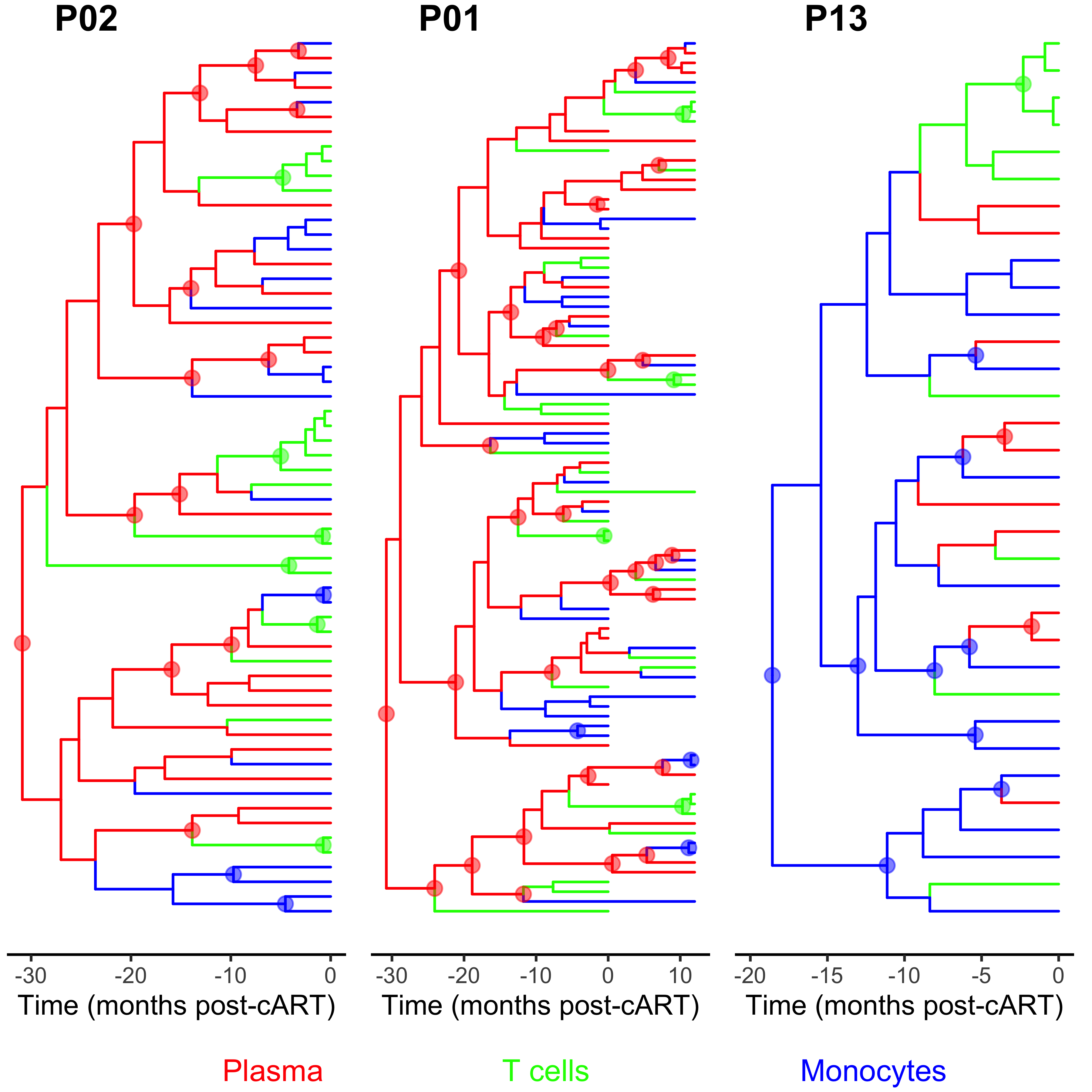

Supplement: veaa005_Supplementary_Data [file veaa005_supplementary_data.zip › FigS10.png]

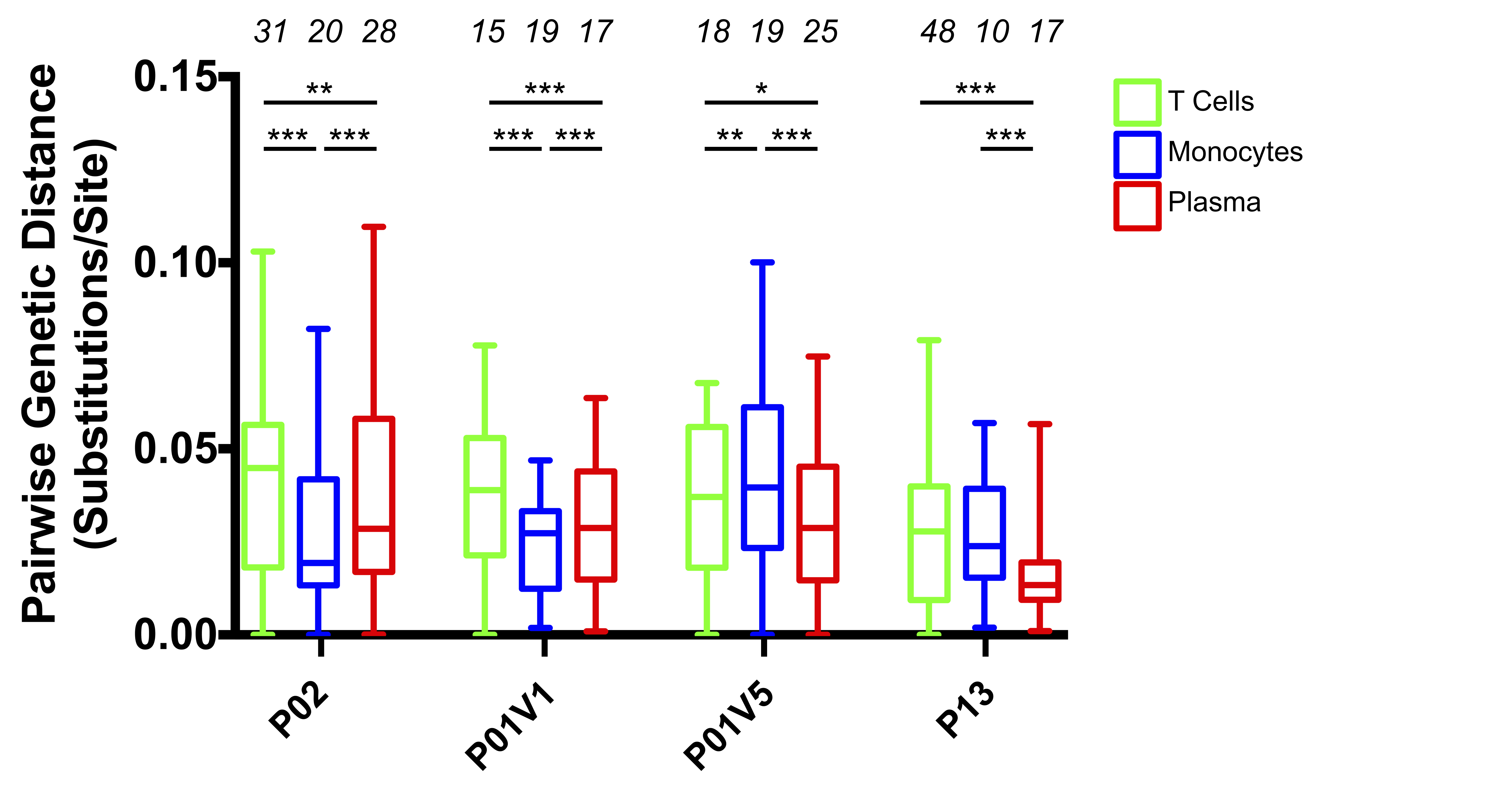

Supplement: veaa005_Supplementary_Data [file veaa005_supplementary_data.zip › FigS11.png]

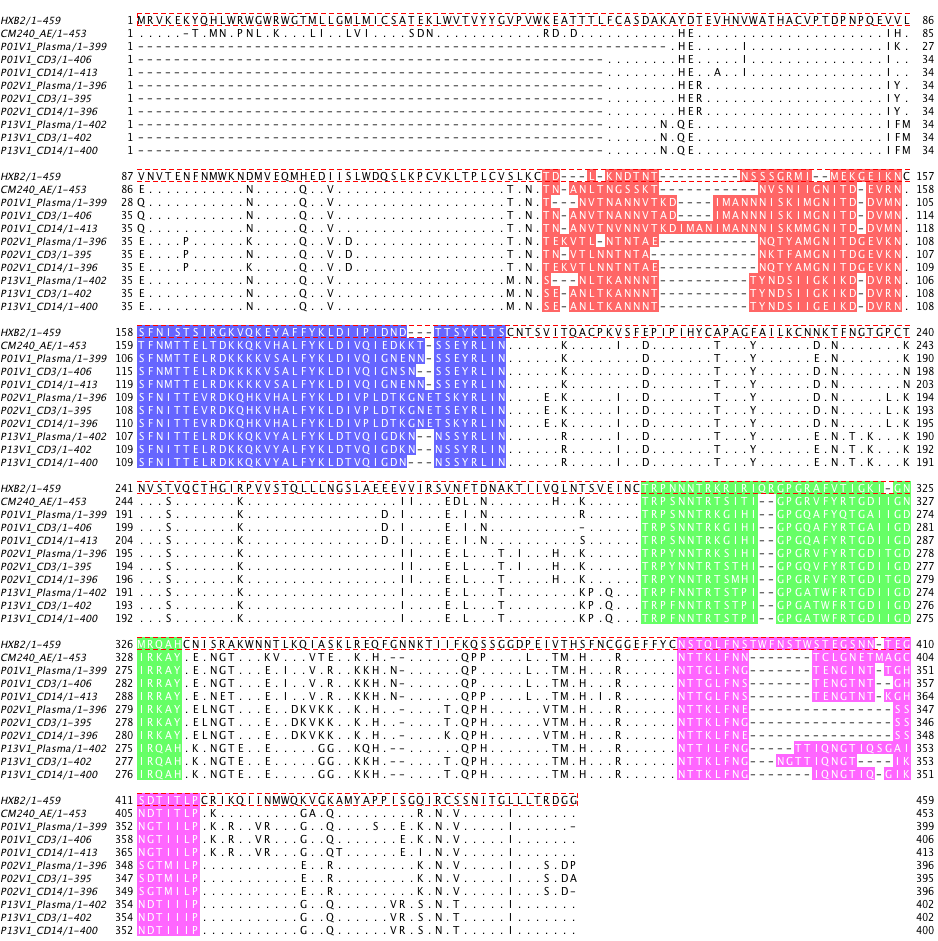

Supplement: veaa005_Supplementary_Data [file veaa005_supplementary_data.zip › FigS12.png]

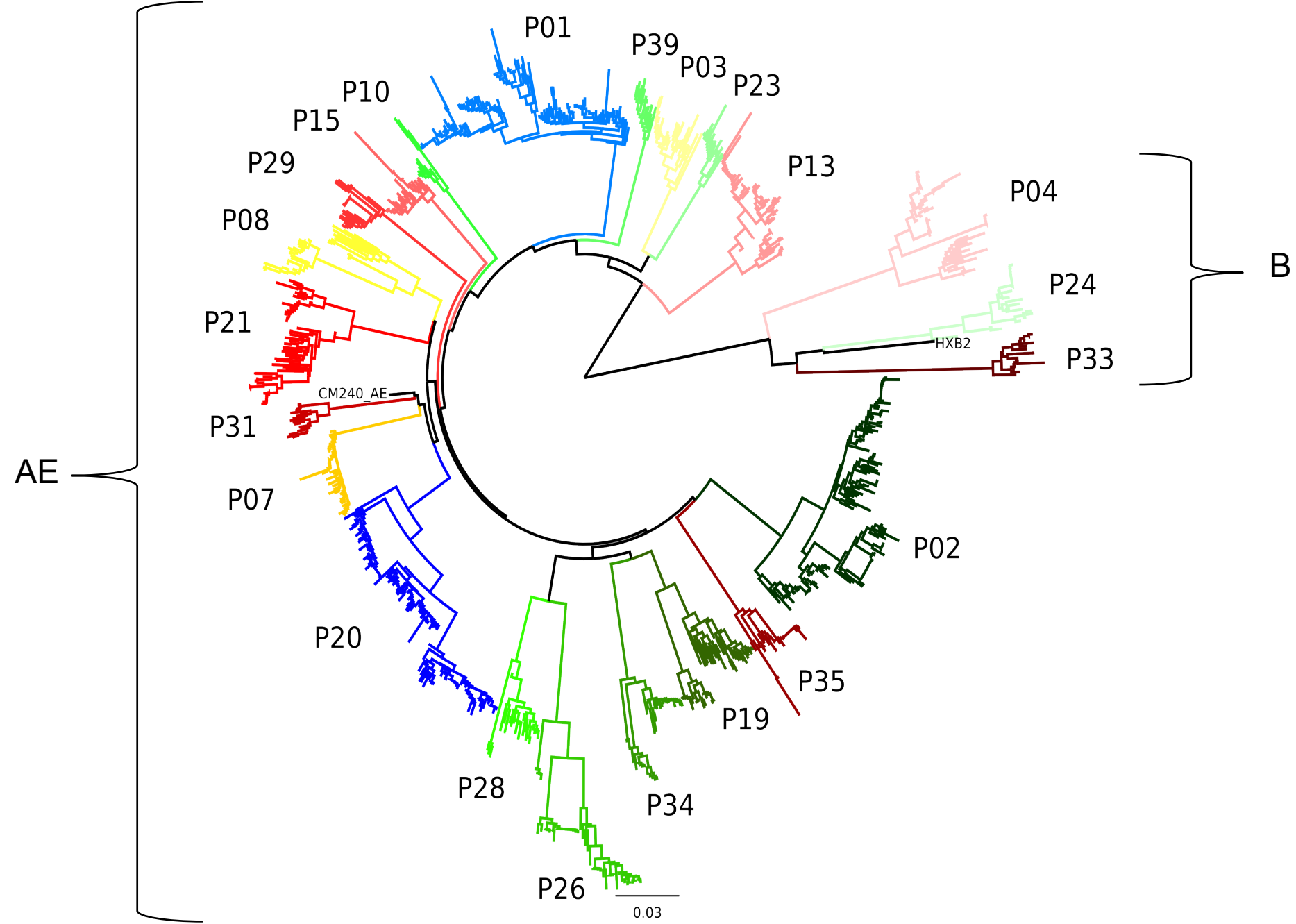

Supplement: veaa005_Supplementary_Data [file veaa005_supplementary_data.zip › FigS1.pdf]

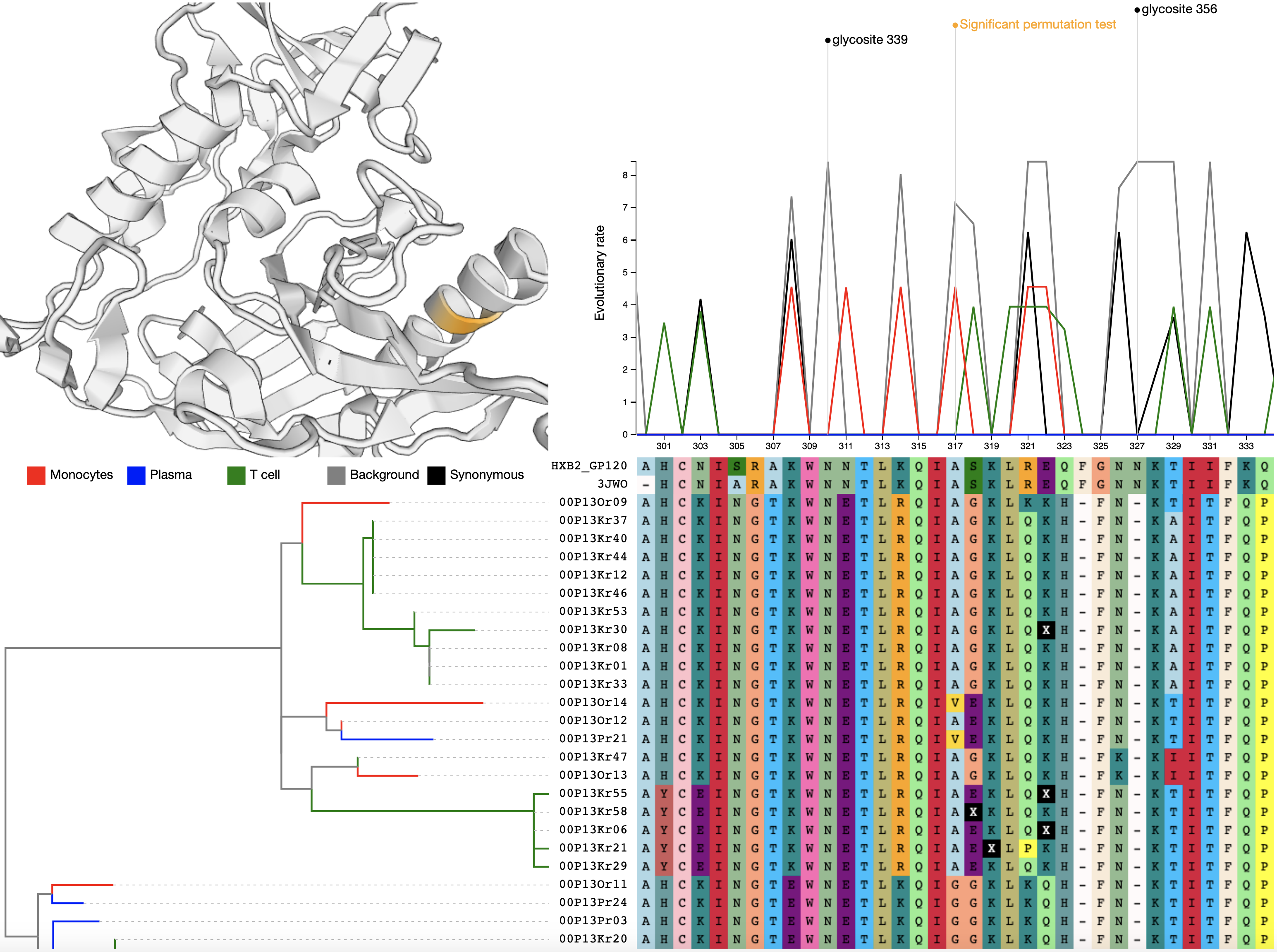

Supplement: veaa005_Supplementary_Data [file veaa005_supplementary_data.zip › FigS2.png]

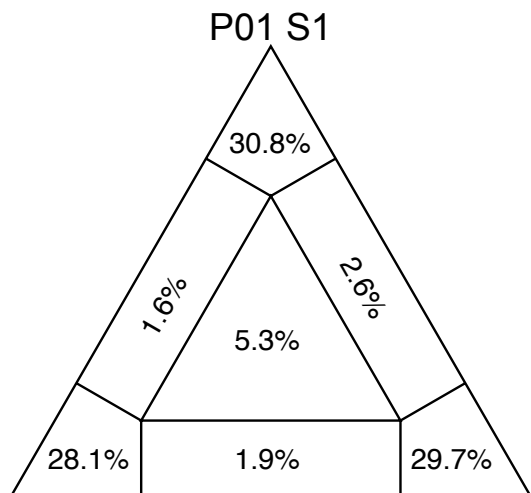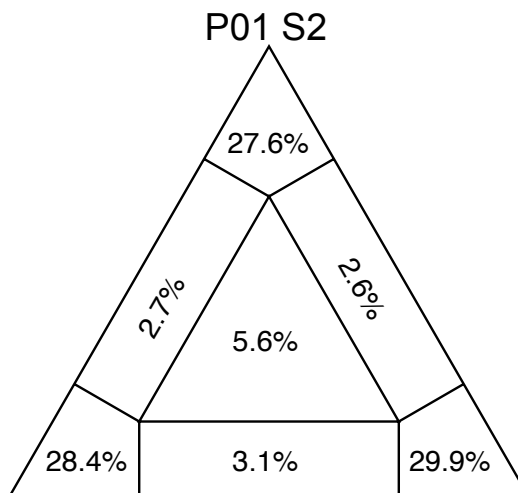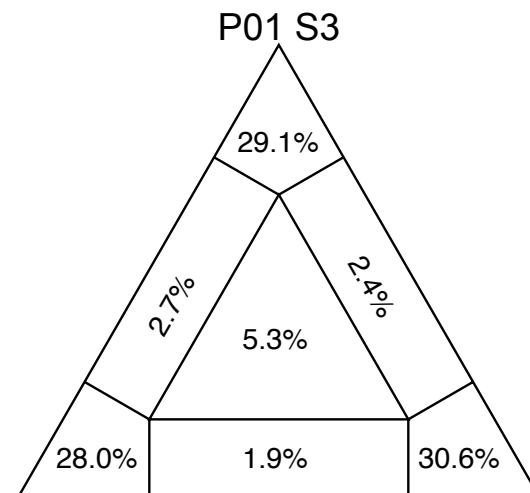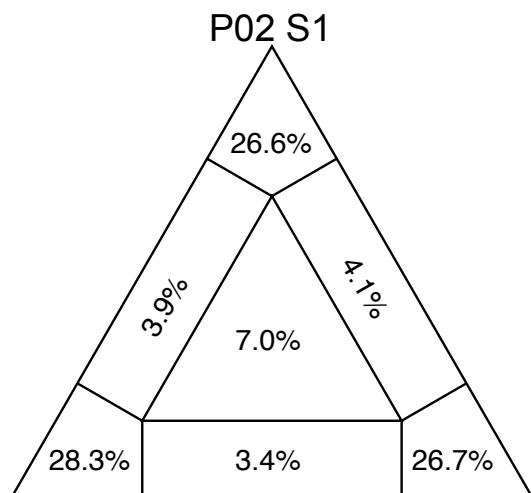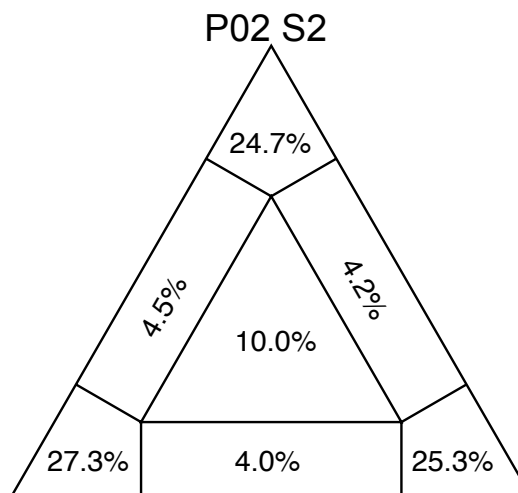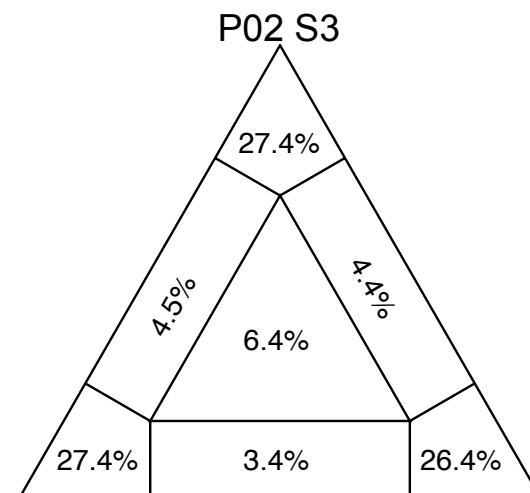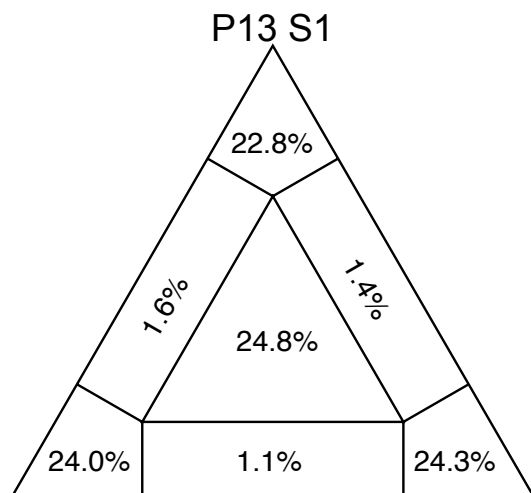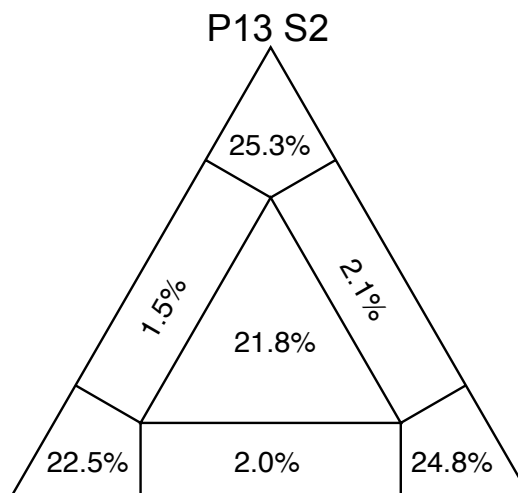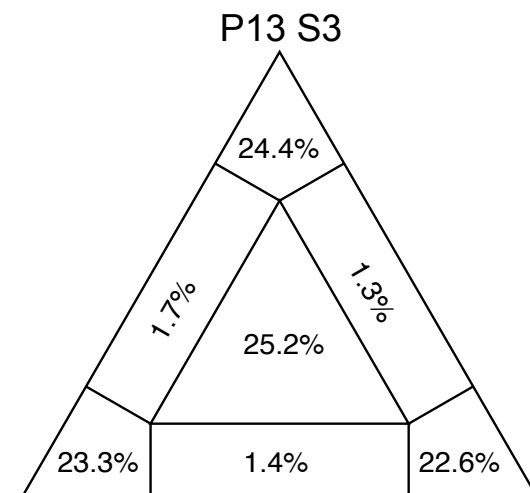

Supplement: veaa005_Supplementary_Data [file veaa005_supplementary_data.zip › FigS3.pdf]

Mean Evolutionary Rate  
(Substitutions/Site/Month)

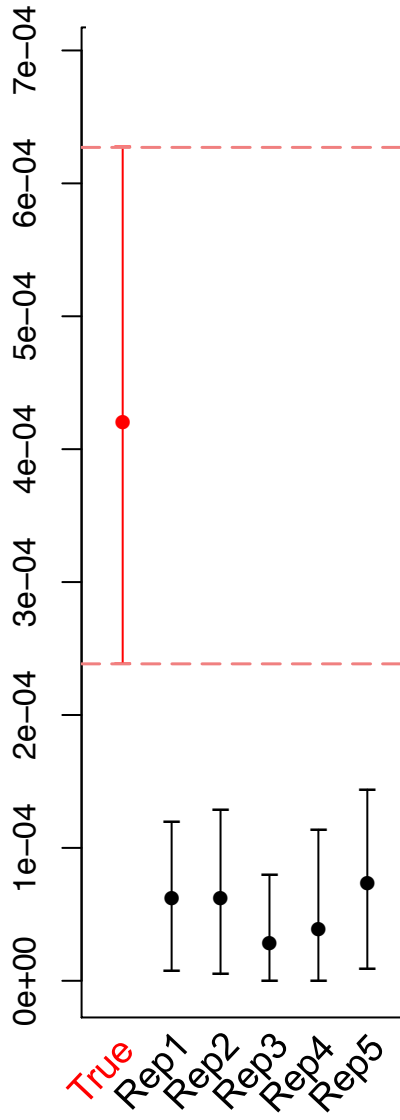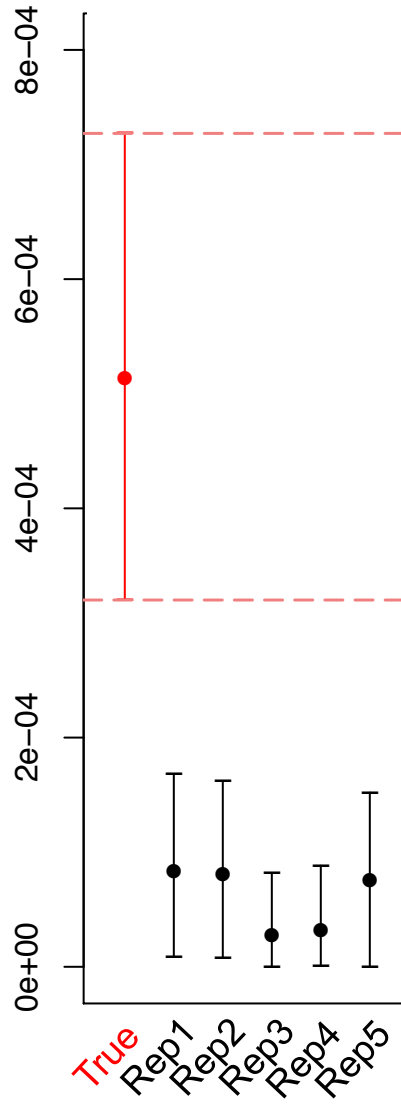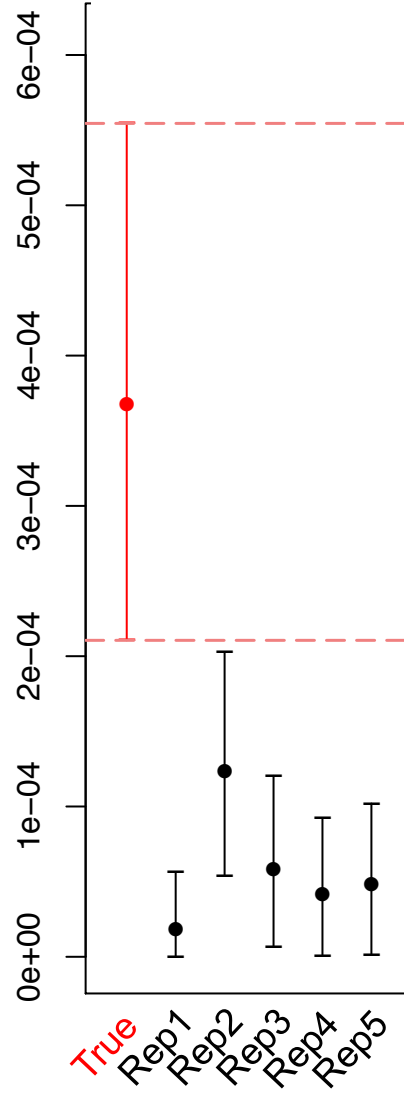

Supplement: veaa005_Supplementary_Data [file veaa005_supplementary_data.zip › FigS4.pdf]

# Gating Strategy for Monocytes Sorting

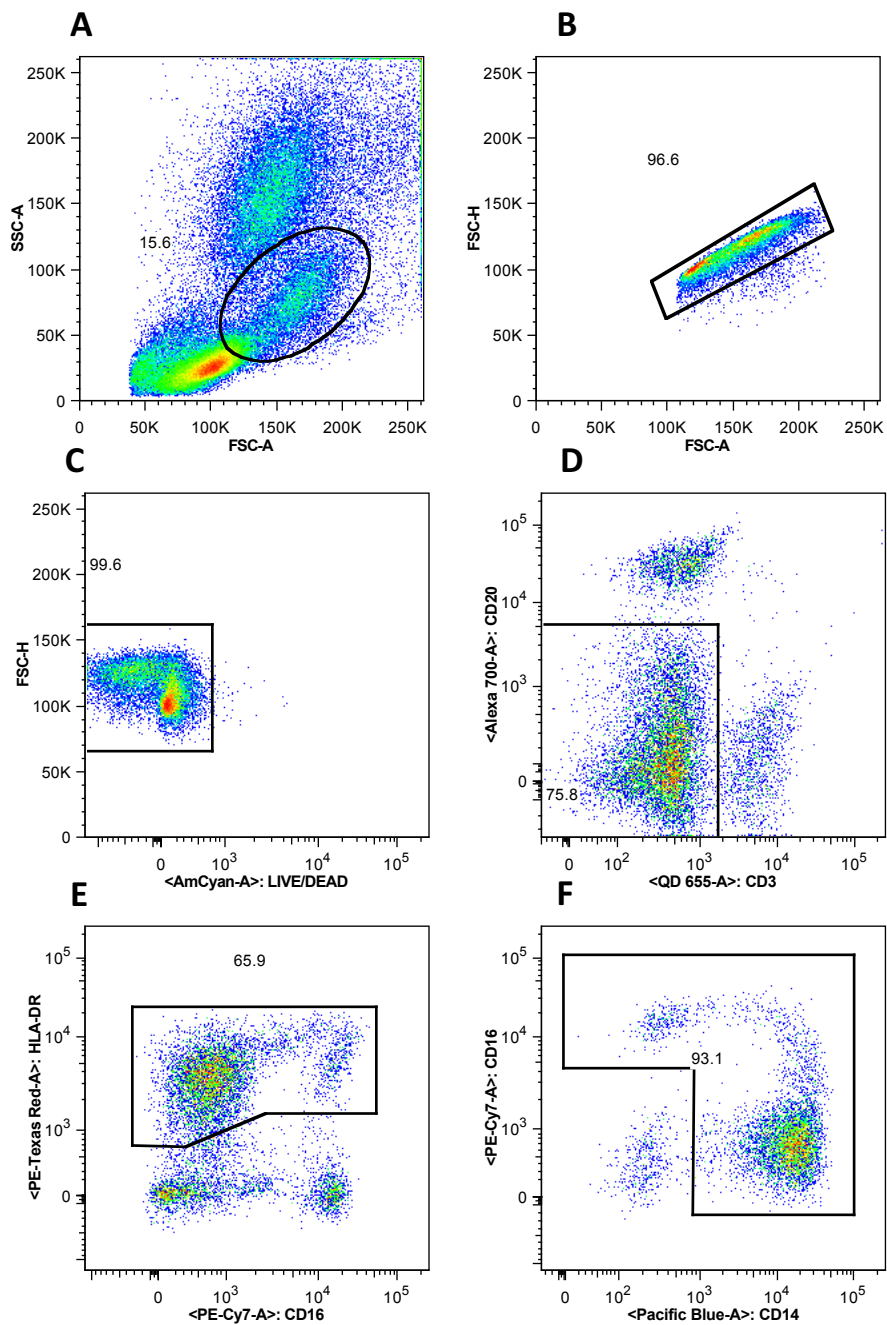

# Post Sort data (ungated)

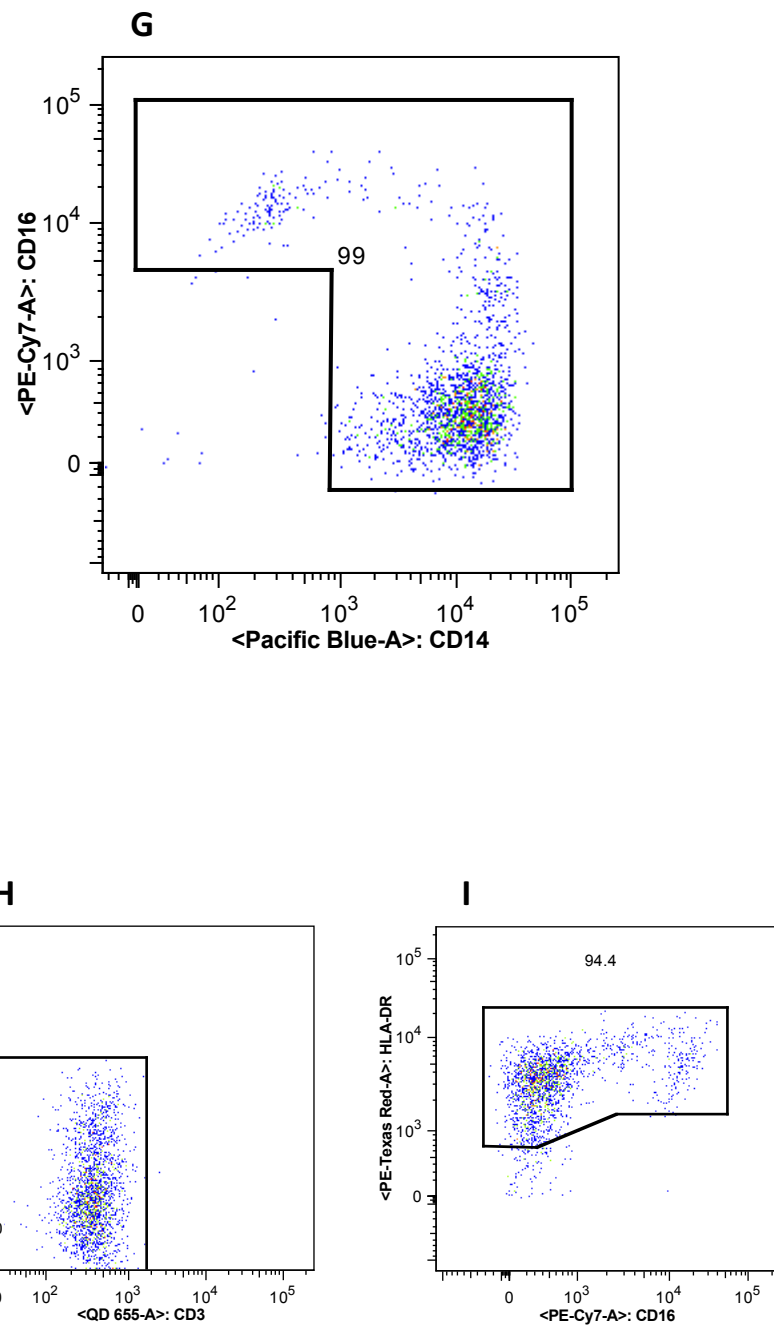

Supplement: veaa005_Supplementary_Data [file veaa005_supplementary_data.zip › FigS5.pdf]

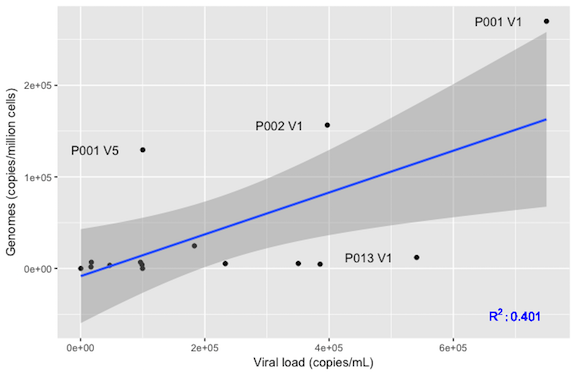

Supplement: veaa005_Supplementary_Data [file veaa005_supplementary_data.zip › FigS7.png]

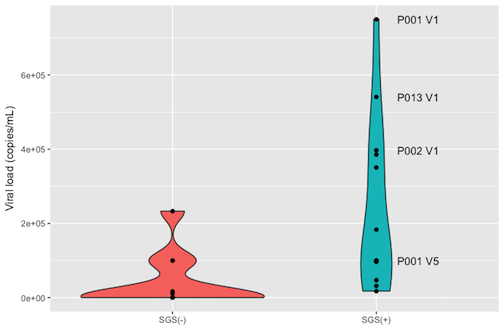

Supplement: veaa005_Supplementary_Data [file veaa005_supplementary_data.zip › FigS8.png]
