# Supplementary material for: Phyloanatomic characterization of the distinct T cell and monocyte contributions to the peripheral blood HIV population within the host
Source: Virus Evol. 2020 Apr 27;6(1):veaa005. doi: 10.1093/ve/veaa005 (PMC7185683; doi:10.1093/ve/veaa005)
Supplement: veaa005_Supplementary_Data [file FigS6.pdf › FigS6.pdf]

	0 MONTHS			3 MONTHS			6 MONTHS			9 MONTHS			12 MONTHS		
PATIENT	PLASMA	T CELL	MONO	PLASMA	T CELL	MONO	PLASMA	T CELL	MONO	PLASMA	T CELL	MONO	PLASMA	T CELL	MONO
P01	17	15	19				15			21			25	18	19
P02	28	31	20				28			32					
P03	19	14	3												
P04	23	21	3												
P07	18	30	2												
P08	21	17													
P10	12														
P13	17	48	10												
P15	27		5												
P19	18	28	3												
P20	22			31		2	19			17	13		35		
P21	28	17	3				8			7					
P23	19														
P24	12	12													
P26	21	25													
P28	25														
P29	13	9	1												
P31	18														
P33	15														
P34	34														
P35	27														
P39	16														
